# Real-World Integration of a Sepsis Deep Learning Technology Into Routine Clinical Care: Implementation Study

**DOI:** 10.2196/15182

**Published:** 2020-07-15

**Authors:** Mark P Sendak, William Ratliff, Dina Sarro, Elizabeth Alderton, Joseph Futoma, Michael Gao, Marshall Nichols, Mike Revoir, Faraz Yashar, Corinne Miller, Kelly Kester, Sahil Sandhu, Kristin Corey, Nathan Brajer, Christelle Tan, Anthony Lin, Tres Brown, Susan Engelbosch, Kevin Anstrom, Madeleine Clare Elish, Katherine Heller, Rebecca Donohoe, Jason Theiling, Eric Poon, Suresh Balu, Armando Bedoya, Cara O'Brien

**Affiliations:** 1 Duke Institute for Health Innovation Durham, NC United States; 2 Duke University Hospital Durham, NC United States; 3 Department of Statistics Duke University Durham, NC United States; 4 John A Paulson School of Engineering and Applied Sciences Harvard University Cambridge, MA United States; 5 Duke University Durham, NC United States; 6 Duke University School of Medicine Durham, NC United States; 7 Duke Health Technology Solutions Durham, NC United States; 8 Duke Clinical Research Institute Durham, NC United States; 9 Data & Society New York, NY United States; 10 Google Mountain View, CA United States; 11 Division of Emergency Medicine Duke University School of Medicine Durham, NC United States; 12 Department of Medicine Duke University School of Medicine Durham, NC United States; 13 Division of Pulmonary, Allergy, and Critical Care Medicine Duke University School of Medicine Durham, NC United States

**Keywords:** machine learning, translational medicine, sepsis, innovation, organizational, change, deep learning

## Abstract

**Background:**

Successful integrations of machine learning into routine clinical care are exceedingly rare, and barriers to its adoption are poorly characterized in the literature.

**Objective:**

This study aims to report a quality improvement effort to integrate a deep learning sepsis detection and management platform, Sepsis Watch, into routine clinical care.

**Methods:**

In 2016, a multidisciplinary team consisting of statisticians, data scientists, data engineers, and clinicians was assembled by the leadership of an academic health system to radically improve the detection and treatment of sepsis. This report of the quality improvement effort follows the learning health system framework to describe the problem assessment, design, development, implementation, and evaluation plan of Sepsis Watch.

**Results:**

Sepsis Watch was successfully integrated into routine clinical care and reshaped how local machine learning projects are executed. Frontline clinical staff were highly engaged in the design and development of the workflow, machine learning model, and application. Novel machine learning methods were developed to detect sepsis early, and implementation of the model required robust infrastructure. Significant investment was required to align stakeholders, develop trusting relationships, define roles and responsibilities, and to train frontline staff, leading to the establishment of 3 partnerships with internal and external research groups to evaluate Sepsis Watch.

**Conclusions:**

Machine learning models are commonly developed to enhance clinical decision making, but successful integrations of machine learning into routine clinical care are rare. Although there is no playbook for integrating deep learning into clinical care, learnings from the Sepsis Watch integration can inform efforts to develop machine learning technologies at other health care delivery systems.

## Introduction

### Background

Technologies that digitize and harness massive amounts of data paired with the alignment of research and clinical care are transforming health care. Machine learning, a set of statistical methods optimized for prediction on new observations, is central to this transformation [1]. Although the translational pathway for prognostic models is well characterized, few machine learning models are externally validated or evaluated in clinical practice [2]. Isolated efforts demonstrating the clinical impact of previously validated technologies show the potential of machine learning in health care [3,4]. However, significant challenges remain for machine learning technologies to become fully embedded within standard operations of health care delivery systems [5].

Machine learning has been rapidly adopted in the biomedical sciences to enhance predictive, prognostic, and diagnostic methods. However, numerous technical and clinical barriers to its adoption persist. First, electronic health records (EHRs) often do not have native functionality to integrate complex machine learning models. Significant investment in infrastructure is required [6-8]. Second, even after a model is initially implemented, machine learning models can incur substantial ongoing maintenance costs [9-11]. Third, although some health systems do build and integrate home-grown machine learning solutions [12,13], that effort is often outsourced to research teams or technology vendors [5]. This divide between operations and model implementation and maintenance presents additional challenges, as “engineering ownership of the input signal is separate from the engineering ownership of the model that consumes it [10].” Finally, many models are not effectively integrated into clinical workflows in a fashion that improves clinical care or outcomes [14].

### Sepsis Watch

Here, we delve into the details of how a health system integrated the first full-scale deep learning technology into routine clinical care. An innovation group spent over two years with partners across the organization to launch a deep learning solution, Sepsis Watch, on November 5, 2018. Sepsis Watch is a sepsis detection and management platform used by clinicians to improve compliance with recommended treatment guidelines for sepsis and thereby improve patient outcomes. Although Sepsis Watch is an instance of machine learning clinical decision support (CDS), deep learning systems do pose implementation challenges beyond traditional CDS, as detailed elsewhere [14-16]. In particular, new mechanisms of trust and accountability must be developed to ensure that the systems are safe and reliable [17,18]. In Table 1, we present the 8 steps required to integrate Sepsis Watch into routine care delivery successfully. We draw upon lessons from the learning health system framework and previously described best practices for responsible machine learning in health care [19,20]. The aim of this manuscript is to describe each step in detail and highlight learnings that can inform related efforts at other organizations.

**Table 1:** Steps for integrating machine learning into clinical care. The table includes definitions for the various steps and example tasks and deliverables during the step.

Step in the process	Definition	Example tasks and milestones
Problem assessment	Understand the root cause of the problem, the magnitude of the problem, where the problem is felt most acutely, who is best positioned to address the problem, and what changes need to occur to empower someone to address the problem	Data analysis to understand the magnitude, setting, and timing of the problem Observe frontline staff in clinical settings where the problem occurs Interview a broad group of stakeholders to understand complexities in addressing the problem
Internal and external scans of solutions and workflows	Perform due diligence on internal and external tools that attempt to address the problem	Evaluate technologies and workflows available through current information technology supplier relationships Evaluate technologies on the market sold by external vendors Interview internal stakeholders who have previously attempted to solve the problem
Clinical workflow design	Design clinical workflow that integrates new technology to address the problem	Gather requirements from frontline staff and leadership Iterate on workflow designs with frontline staff Identify constraints (eg, time and effort) to ensure that the end user is able to use the technology effectively
Model and infrastructure design	Design machine learning model and accompanying infrastructure to ensure that the technology can effectively be integrated into clinical workflows	Identify a set of input features used by the model to address the problem, making sure to incorporate clinical domain expertise and prior literature Design infrastructure to support clinical decisions in a timely, actionable manner Identify performance metrics and goals that are most important and relevant to stakeholders and end-users
Clinical workflow application development	Develop the clinical workflow application and integrations with other technologies	Develop user interface and user experience Integrate with electronic health record to access the required data at the required latency Prototype workflow application with end users
Model and infrastructure development	Develop the machine learning model and infrastructure required to implement model, including integrations with other technologies	Develop and validate the machine learning model on retrospective data Validate the machine learning model and infrastructure on prospective *silent period* launch
Implementation, change management, and governance	Implement the machine learning model with accompanying education, communication, and governance to ensure accountability and successful adoption	Establish a governance committee with agreed-upon tasks mission Develop training material to ensure end users effectively use the new technology Communicate broadly about the technology implementation and roles and responsibilities
Evaluation plan and partnerships	Prespecify evaluation plan, target goals, and safety and efficacy monitoring	Develop internal and external partnerships to ensure rigorous evaluation Register clinical trial

## Methods

### Problem Assessment

In October 2015, an interdisciplinary team of frontline clinicians at Duke Health proposed an innovation project to improve early detection of sepsis. With strong support from senior leadership, this project was launched in April 2016. The pilot project began at the academic flagship hospital, Duke University Hospital (DUH), and if the pilot yielded successful results, it would be expanded to 2 Duke Health community hospitals. Earlier attempts to implement CDS to improve timely detection and response to inpatient deterioration, including sepsis, caused significant alarm fatigue and did not improve clinical outcomes [21]. The Centers for Medicare and Medicaid Services (CMS) SEP-1 measure calculates compliance with 3-hour and 6-hour treatment bundles, and at the time the project began, in 2016, SEP-1 was progressing toward public reporting [22]. SEP-1 performance at DUH was poor, and clinical leaders found that although patients often received individual items of the sepsis bundle, follow-up items at 3 and 6 hours were often not completed.

Health system leaders wanted to reimagine how data and technology could be effectively utilized to both detect sepsis and coordinate care to ensure the completion of recommended bundles. The team adopted computable sepsis criteria aligned with the CMS SEP-1 measure and quality improvement efforts at peer institutions, specified in Multimedia Appendix 1 [23,24]. Specific time windows to consider for each data element along with thresholds were decided upon by an interdisciplinary team of clinicians. Using these criteria, an analysis of DUH admissions data revealed that 55% and 68% of sepsis occurred within 12 hours and 24 hours, respectively, after presentation to the DUH emergency department (ED). Similarly, chart reviews of terminal hospitalizations involving sepsis found that over 70% of sepsis presented in the ED [25]. Hence, the initial clinical integration focused on improving sepsis detection and management among adults in the DUH ED. Table 2 displays characteristics of adult patients presenting between March and August 2018 to the site of the first integration, the DUH ED.

### Internal and External Scans of Solutions and Workflows

In 2016, efforts to predict sepsis largely used the Medical Information Mart for Intensive Care [26] and restricted focus to just intensive care units (ICUs) [23,27,28]. At that time, there were successful reports of sepsis CDS algorithms [29], but there was no validated machine learning method to predict sepsis among adult patients presenting to the ED. A model specific to the ED predicted inpatient mortality among patients already meeting sepsis criteria [30]. The newly published quick Sequential Organ Failure Assessment (qSOFA) was recommended to identify patients at risk of poor outcomes because of sepsis [31]. However, qSOFA was not adopted by CMS for the SEP-1 core measure and does not accurately identify patients at risk of developing sepsis [24,32]. Considering the lack of an available, validated model to predict sepsis in the ED at that time, an interdisciplinary team of clinicians proposed to develop a novel machine learning model using local data.

The success of the innovation pilot depended on the rapid translation of model output to clinical action. The prior CDS implementation indicated that alert-fatigued frontline staff might not be the right personnel to receive alerts. The clinical team agreed that the rapid response team (RRT) nurses within DUH were best suited to triage patient alerts to manage sepsis proactively. RRTs significantly reduce time to medical resuscitation and escalation of care and, for sepsis specifically, improve the delivery of care bundles and outcomes [33-36]. The Sepsis Watch workflow needed to account for RRT nurses being mobile, caring for patients throughout the hospital, and needing to rapidly switch tasks to attend to urgent clinical duties.

### Clinical Workflow Design

**Figure 1:**
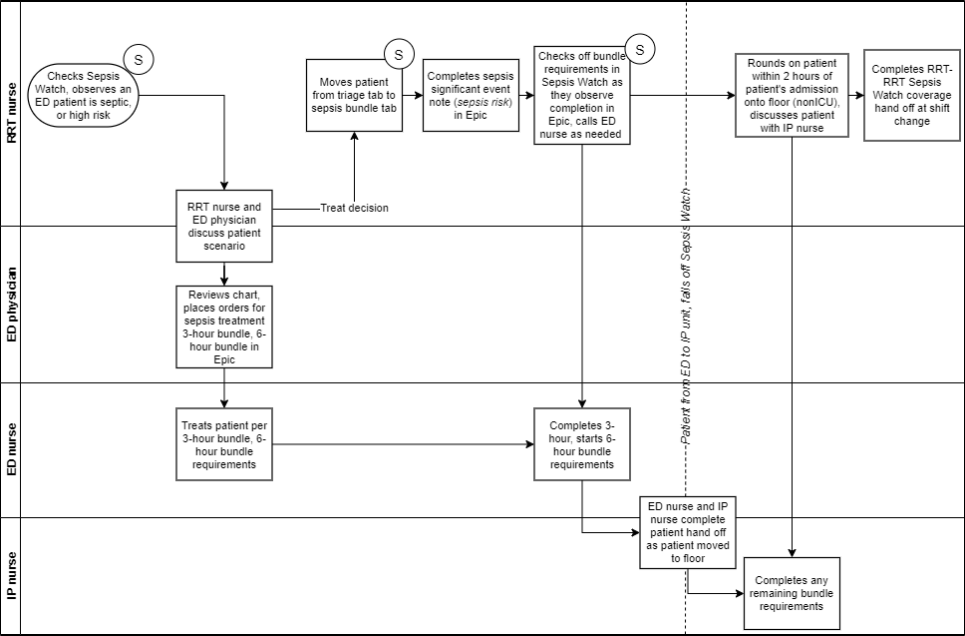
Sepsis Watch swimlane diagram.

During the design phase, a transdisciplinary team of data scientists, statisticians, hospitalists, intensivists, ED clinicians, RRT nurses, and information technology leaders was assembled. The team designed the model and workflow concurrently and invested significant effort into gathering requirements from various stakeholders before writing code. Sepsis Watch was designed to be an overlay on top of existing clinical care within the ED, in contrast to the management of stroke and ST-elevation myocardial infarction, which require specialized teams to comanage patients alongside ED staff. For Sepsis Watch, all individual diagnostic and treatment actions are executed by the ED staff. Because of this distinction, the term *code sepsis* was avoided when describing Sepsis Watch. This workflow required a clear definition of roles and responsibilities across the RRT and ED clinical teams. Figure 1 presents the Sepsis Watch workflow. The RRT nurse triages patients at risk of sepsis using Sepsis Watch and communicates with ED clinicians about recommended treatment bundles. For patients who are confirmed to need treatment for sepsis, the RRT nurse enters a templated *significant event note* into the EHR. This note is meant to be a record of the RRT nurse evaluation and documentation for the admitting attending to carry out any remaining bundle items. The RRT nurse combined the use of Sepsis Watch with other clinical responsibilities across the hospital and communicated with ED clinicians telephonically. The Sepsis Watch user interface was carefully designed to accommodate tablet and mobile phone use.

**Table 2:** Cohort demographics for adults presenting to the Duke University Hospital emergency department between March 1, 2018, and August 31, 2018 (N=39,918).

Baseline characteristics of cohort			Values
Age (years), mean (SD)			50.41 (19.58)
Sex (male), n (%)			18,324 (45.90)
**Admission source,** **n (%)**			
	Home or non–health care facility	34,892 (87.41)	
	Transfer from hospital	2887 (7.23)	
	Missing or other	2139 (5.36)	
**Admission type,** **n (%)**			
	Elective	5617 (14.07)	
	Emergency	30,099 (75.40)	
	Urgent	4160 (10.42)	
**Race,** **n (%)**			
	Black or African American	15,858 (39.73)	
	Caucasian or white	19,737 (49.44)	
	Missing or other	4323 (10.83)	
**Ethnicity,** **n (%)**			
	Not Hispanic/Latino	36,505 (91.45)	
	Hispanic/Latino	2352 (5.89)	
	Missing/other	1061 (2.66)	
**Comorbidities,** **n (%)**			
	Congestive heart failure	3349 (8.39)	
	Peripheral vascular disease	1685 (4.22)	
	Hypertension	11,934 (29.90)	
	Pulmonary circulation disorders	4063 (10.18)	
	Diabetes mellitus without chronic complications	2918 (7.31)	
	Solid tumor without metastasis	3949 (9.89)	
	Obesity	3225 (8.08)	
	Fluid and electrolyte disorders	5890 (14.76)	
	Anemia	3340 (8.37)	
	Depression	2733 (6.85)	
**Prior sepsis encounters in the past year,** **n (%)**			
	0	39,002 (97.71)	
	1	682 (1.71)	
	2 to 5	234 (0.59)	
**Septic,** **n (%)**			2593 (6.50)
	Emergency department	1377 53.10)	
	ICU^a^	468 (18.05)	
	General floor	602 (23.22)	
	Surgery	226 (8.72)	
Overall rate of encounters that resulted in an admission, n (%)			18,620 (46.65)
Overall rate of ICU admission, n (%)			4668 (11.69)
Overall length of stay (hours), median (25% percentile, 75% percentile)			13.72 (5.11, 90.46)

^a^ICU: intensive care unit.

### Model and Infrastructure Design

The machine learning model was designed to detect sepsis early enough to provide clinicians time to confirm the diagnosis and complete CMS SEP-1 bundles. Model input features were both static (eg, prehospital patient comorbidities, patient demographics, and encounter details), and dynamic (eg, medication administrations, laboratory results, and vital measurements). Multimedia Appendix 1 lists all model features. The model was designed to perform well on adult patients who present to the ED from the time of ED triage through admission until time of death, discharge from the hospital, or admission to an ICU. The model was not trained to detect sepsis in the ICU setting. Patients admitted directly to surgery were excluded from model development. Similar to other sepsis prediction models [12], model inputs included both patient physiology (eg, vital sign measurements) and clinical interventions potentially prompted by suspicion of sepsis (eg, administration of antibiotics, measurement of serum lactate). In prior work, we demonstrated that inclusion of clinical interventions, such as indicators for whether or not a measurement or medication administration occurred in any given hour, improved model performance [37,38]. The practice of including clinical interventions as model inputs has been recommended for models built to be integrated into clinical practice [11].

Clinical leaders prioritized positive predictive value as a performance measure and were willing to trade-off model interpretability for performance gains. Model interpretability was low priority because of the many causes of sepsis, and treatment protocols are largely agnostic to cause. Sepsis Watch was designed to use the machine learning model to alert clinicians to evaluate patients further. Clinicians were instructed to put the model output into context with other relevant information to confirm or dismiss a sepsis diagnosis. The machine learning model did not drive clinical care in a standalone manner. The team worked closely with regulatory officials to ensure that Sepsis Watch qualified as CDS and was not a diagnostic medical device.

The team collaborated with technical and clinical stakeholders to define system requirements. The machine learning model needed to update the risk of sepsis for all patients every hour, whereas patients who met sepsis criteria needed to be identified every 5 minutes. Epic Web services needed to be built to allow Sepsis Watch to extract data from Epic every 5 minutes. The data are nurse-verified, and there are currently no interfaces to other monitors or data streams. The innovation pilot initially focused on an ED with approximately 200 visits per day and needed to be scalable to 1500 inpatient beds across the health system. The infrastructure was fully automated, fault-tolerant, parallelized, and run on on-premise computing infrastructure. During the 6-month pilot, the system uptime was 99.34%, with 1 instance of planned patching, 2 instances of the Web application being temporarily unavailable, and 1 instance of data not being updated. Sepsis Watch application code was to enhance portability and to scale across on-premise or cloud virtual environments while also improving reproducibility, security, and ease of management. A database storing risk scores, time of sepsis, and information relevant to sepsis supported the Sepsis Watch Web application.

### Clinical Workflow Application Development

**Figure 2:**
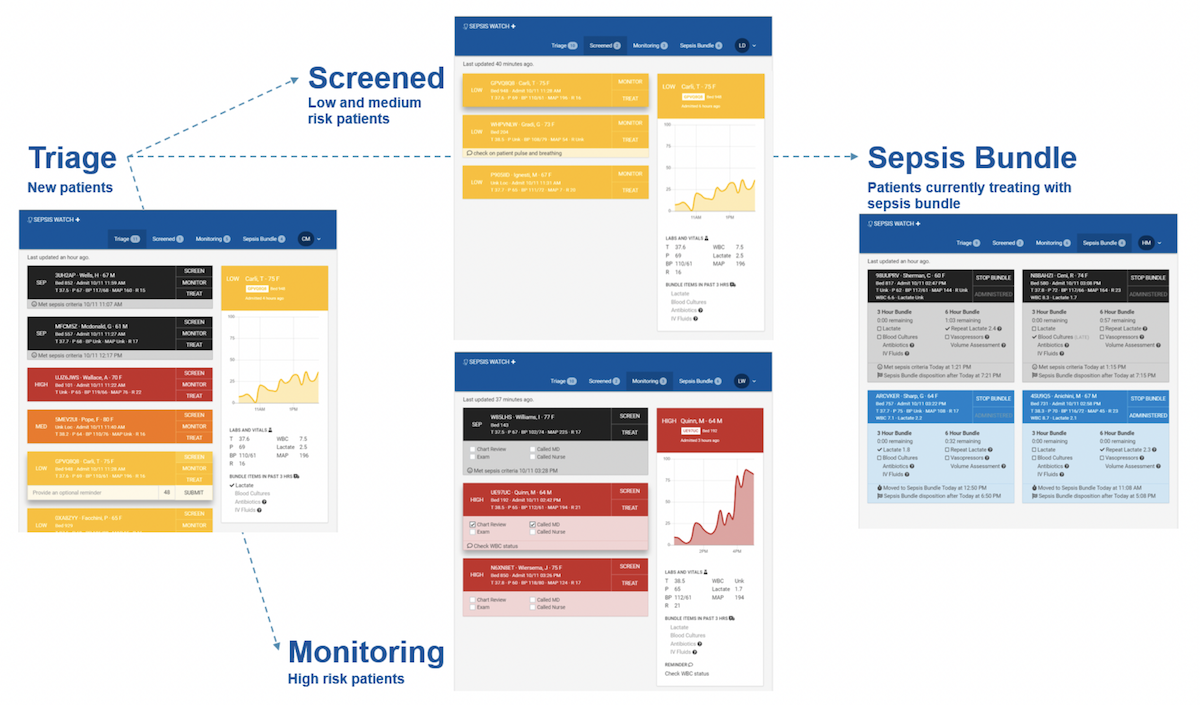
Sepsis Watch user interface.

After gathering requirements and iterating on designs of the workflow and model, development began in parallel on the Sepsis Watch Web application and a custom deep learning model. The Sepsis Watch Web application was developed in close collaboration with frontline staff. User interface designers repeatedly met with RRT nurses to iterate on functions, information, control, and visual components of the design. The first version contained 3 lists of patients: *Screening*, *Watchlist*, and *Treatment*. There was a 12-hour *snooze* state that prevented patients from being presented on the application. A second version removed the *snooze* state and included 4 lists of patients: *Triage*, *Screened*, *Monitoring*, and *Treatment*. This version ensured that all patients were visible at all times. The *Triage* page was the first point of entry for all patients presenting to the ED. Patients not requiring further evaluation were placed on the *Screened* page, whereas patients requiring further evaluation were placed on the *Monitoring* page. The *Treatment* page tracked completion of 3- and 6-hour sepsis bundle items for patients receiving treatment. Figure 2 displays screenshots of the application pages. Sepsis Watch was originally conceptualized as a dashboard to display model output; however, feedback and iterations led to the development of a highly interactive workflow management solution. Patients who met sepsis criteria were displayed in black colored cards, whereas patients at high risk of sepsis were displayed in red-colored cards. RRT nurses called ED physicians to discuss every patient with sepsis or at high risk of sepsis. No patient was placed on the *Treatment* page without independent review and confirmation by the attending physician.

### Model and Infrastructure Development

A novel machine learning method that combined multitask Gaussian processes (MGPs) and recurrent neural networks (RNNs) was developed to detect sepsis early [37,38]. RNNs are a type of deep learning configured to ingest time series data and are ideally suited to combine static and dynamic features of hospital encounters that vary in length [39,40]. The MGP learned distributions of continuous functions for each dynamic variable. Every hour, dynamic features sampled from the MGP were combined with static features and fed into the RNN to generate a risk of sepsis between 0 and 1. A separate set of scripts was optimized to run every 5 min to identify patients who meet sepsis criteria [41].

Our transdisciplinary team collaborated with Epic Systems to identify an optimal path for accessing clinical data in real time and integrating Sepsis Watch into the production system. A combination of off-the-shelf and custom-built Web services was utilized so that new patient information could be pulled every 5 min and stored in an external database. Given that the model ingests data from a variety of domains, 2 tools were developed to monitor Sepsis Watch. First, a Web service monitoring system and Web page was built to ensure that real-time integrations with Epic functioned appropriately. Second, a model monitoring system and Web page was built to display daily and weekly counts and mean values of model inputs and outputs.

## Results

### Implementation, Change Management, and Governance

A 3-month *silent period* was implemented before launch, during which Sepsis Watch first interacted with real-time data. A final round of data mapping was performed with clinical validation to reconcile changes in data formatting, followed by end-to-end testing of the model, data pipeline, user interface, and workflow. The first version of the model implemented the RNN without the MGP with minimal reduction in performance, reflecting practical trade-offs often made between model performance and engineering effort [42]. Thresholds were set to optimize positive predictive value and the number of alerts. Up to 4 high-risk alerts per hour were agreed upon as ideal volume for a single RRT nurse user. Clinical leaders reviewed 50 high-risk cases with a 72-hour delay to validate the threshold. Sepsis Watch accounts were created for clinical leaders to validate model output and test the workflow. Clinical leaders were instructed to contact inpatient teams if an observed patient needed immediate action. On an average day, about 14 patients met sepsis criteria, and about 7 patients were at high risk of sepsis.

Go-Live preparations focused on ensuring effective adoption and integration of Sepsis Watch into clinical care. Before Sepsis Watch, RRT nurses had minimal interaction with ED physicians. For Sepsis Watch to have the desired impact, these 2 roles needed to work closely together. With senior leadership support, regular touchpoints were prioritized to align partners around a unified vision of the workflow and potential impact. In the 4 weeks leading up to Go-Live, nearly a dozen hours per week were spent cultivating relationships and communication channels between roles. Weekly meetings brought together 1 to 2 leaders, each from the RRT nurse, ED nurse, ED physician, and inpatient hospitalist stakeholder groups. End-of-week updates were sent out every Friday, covering progress during the prior week and goals for the upcoming week to keep the team aligned. It was also during this time that the physicians and RRT nurses involved throughout the 2-year design and development process of Sepsis Watch served as crucial clinical champions promoting trust in the technology. In fact, the lead statisticians and developers had minimal interaction with frontline staff during the 4 weeks leading up to Go-Live. Clinicians with no formal information technology role within the health system promoted Sepsis Watch among their peers as a home-grown solution to an important problem within the hospital.

**Figure 3:**
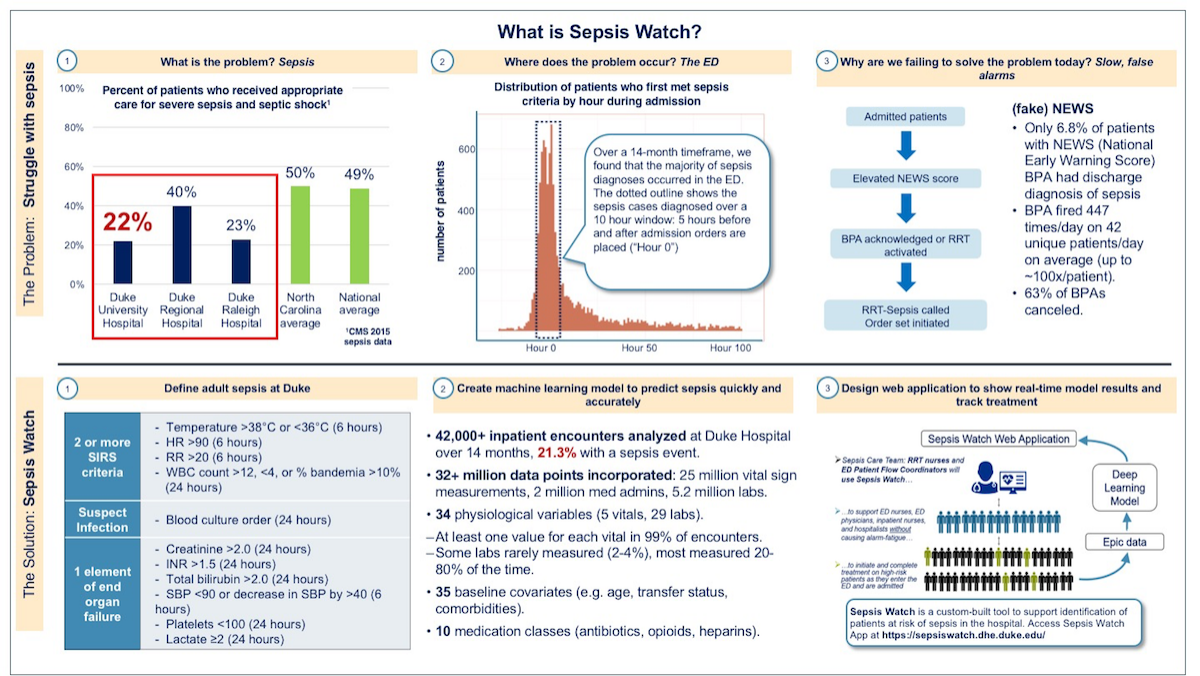
Sepsis Watch training one-page overview.

The next goal to drive adoption was to broadly communicate the change vision and empower action [43,44]. The team began in-person training for RRT nurses, emphasizing the urgent need to improve sepsis care in the ED and the opportunity to improve outcomes with Sepsis Watch. Although all RRT nurses worked in the critical care setting and were familiar with sepsis, training on the diagnostic criteria and treatment for sepsis was included to enhance awareness and understanding. The implementation team walked through the Sepsis Watch workflow with the RRT nurses in detail using a test version of the application populated with synthesized data. RRT nurses then interacted with the test version of the application themselves, iterating through the workflow steps, and asking questions to the implementation team. The in-person training ended with discussions of roles and responsibilities and the identification of various resources available to support frontline staff. Clinical nurse educators helped develop and distribute training content on an intranet webpage. Figure 3 shows a 1-page handout describing Sepsis Watch. This material was also communicated across clinical units through standing meetings and email listservs.

Sepsis Watch launched at 12 PM Eastern Daylight Time on November 5, 2018. The inaugural user was an RRT nurse who helped design the system. The ED medical director briefed the ED physicians to expect phone calls starting at noon. The RRT nurse was equipped with a tablet loaded with a link to the Sepsis Watch application, Epic’s Canto app, the Sepsis Watch training homepage, contact information for all ED clinicians, a map of the ED, and a 2-min survey for submitting application and workflow feedback. The tablet and Sepsis Watch coverage were handed off at the end of each 12-hour shift. The Sepsis Watch Go-Live proceeded smoothly, and the application remained in continuous use by RRT nurses throughout the pilot.

The Sepsis Watch governance committee was created to monitor effectiveness and promote broad-based action. The committee included nursing, physician, and administrative leadership across the ED and inpatient wards. The committee’s 4 primary goals were to (1) promote usage of the Sepsis Watch app, (2) provide comprehensive training and communication on the application and workflow, (3) develop a reporting method to track patient volume and bundle compliance, and (4) plan for postpilot sustainability. Table 3 lists the volume and bundle compliance metrics prioritized by the committee to include in weekly reports. These metrics provided clarity on compliance with specific bundle items, ensured that the volume of alerts was reasonable for a single RRT nurse user, and identified short-term wins to boost momentum. The implementation team sent weekly reports consisting of these metrics to frontline staff, including RRT nurse team members, and the Sepsis Watch governance committee.

**Table 3:** Sepsis Watch governance weekly report metrics.

Metric types^a^	Metrics
Volume	Average number of new patients appearing on the Sepsis Watch Triage tab per day
Volume	Distribution of new patients appearing on the Sepsis Watch Triage tab, by hour of the day
Volume	Median length of time patient remained on the Sepsis Watch Triage tab before being moved to another tab
Volume	Average number of patients moved to the Sepsis Bundle Treatment tab per day
Bundle compliance	3-hour bundle compliance for patients moved to the Sepsis Watch Treatment tab (comprised of antibiotics, lactate, and blood culture 3-hour bundle components). Includes week-by-week performance
Bundle compliance	Antibiotics administration 3-hour compliance for patients moved to the Sepsis Watch Treatment tab. Includes week-by-week performance
Bundle compliance	Serum lactate collected 3-hour compliance for patients moved to the Sepsis Watch Treatment tab. Includes week-by-week performance
Bundle compliance	Blood culture collected 3-hour compliance for patients moved to the Sepsis Watch Treatment tab. Includes week-by-week performance

^a^Metrics were chosen by the Sepsis Watch governance committee to present data for 2 distinct patient cohorts: (1) patients who met Sepsis Watch sepsis criteria and (2) patients who were at high risk for meeting Sepsis Watch sepsis criteria as identified by the model.

### Evaluation Plan and Partnerships

The Sepsis Watch evaluation consisted of continuous improvements to the workflow and user interface based on user feedback, 2 qualitative evaluations of how the technology impacted frontline clinicians, and a clinical and operational impact evaluation to demonstrate safety and efficacy. To enable continuous improvements, frontline staff regularly communicated feedback both indirectly, through a web-based survey that was bookmarked on the tablet, and directly to the team during regularly scheduled meetings or via email. These feedback loops were crucial to improving Sepsis Watch. All proposed changes and adaptations were prioritized and approved by the governance committee. For example, an early workflow change was to have the RRT nurse call the primary ED bedside nurse directly to ensure completion of sepsis treatments (eg, administration of antibiotics that are already ordered). Before this change, the RRT nurse was calling a charge nurse that managed ED intake triage, and the charge nurse was then expected to communicate with ED bedside nurses. We found that rather than centralizing information flow, the information needed to be communicated with the clinicians most directly involved in the care of patients who needed immediate action. Other changes related to improving communication channels included adopting *first call provider* functionality in the EHR to make explicit the covering physician for each patient that the RRT nurse needed to call and improving the layout of the phone number reference list for tablet use.

Similarly, a handful of changes were made to the user interface in the second version of the Sepsis Watch that was pushed out in January 2019. RRT nurses began using a standardized paper template to supplement Sepsis Watch on the tablet. The goal of the update was not to eliminate the need for a paper workflow supplement but to bring functionality that would further enhance efficiency into the application. For example, instead of comments being limited to patient transitions between lists, comments were enabled for all patients, and the character count was increased from 80 to 200. In addition, rather than only flagging sepsis bundle items that are complete (eg, blood culture collection and antibiotic administration), a flag was created for sepsis bundle items that are ordered (eg, blood culture ordered and antibiotic ordered). Before going live, the update was reviewed and approved by both frontline RRT nurses and the governance committee.

All new machine learning implementations have the potential for introducing inequality and bias that is not always clearly visible in numeric data [45-48]. Growing concern about such biases in health care highlighted the need for specialized evaluation of the Sepsis Watch [49-51]. Reflecting a commitment to rigorously study the outcomes and implications of integrating deep learning into clinical care, collaborations were established with 2 social science research institutes, Duke University’s Social Science Research Institute (SSRI) and Data & Society Research Institute (D&S). Furthermore, 2 qualitative studies were designed to investigate the sociocultural dimensions of clinical integration. One evaluation, carried out with SSRI, focused on structured and semistructured interviews of ED physicians and RRT nurses, and the other evaluation, carried out with D&S, focused on observations of ED physicians and RRT nurses. Both studies analyzed Sepsis Watch in the context of organizational change management. These efforts aimed to identify adoption barriers and facilitators, and unintended social consequences and shifting clinical roles and responsibilities. Preliminary analysis of clinician’s perceptions of evidence, trust, and authority in the early phase of development was completed, and several salient findings emerged [52]. First, building trust in the technology required much more than demonstrating model performance on a holdout and temporal validation set to clinicians. Stakeholders were looped in from the very beginning of the project, and it was important for the technology developers not to be or be seen as telling clinicians how to do their work. Second, the team identified the type and extent of evidence that was most salient to each stakeholder group. Although numbers and statistical trends were highlighted to hospital leaders, administrators, and managers, individual patient cases were important to frontline clinicians. This insight led to the development of patient-specific sepsis bundle reports that will go out to physicians and nurses involved in a patient case. Third, the team had to carefully navigate the lines of professional authority that physicians have toward the care of patients. Throughout the design, development, and implementation process, Sepsis Watch was described as a *tool* to support physicians and nurses in the ED, and the term *artificial intelligence* was not used in any communication or presentation.

The clinical impact will be evaluated (ClinicalTrials.gov ID: NCT03655626) after completion of the pilot. The primary outcome for this clinical trial is sepsis treatment bundle compliance. Secondary clinical outcomes include inpatient mortality, ICU requirement, and hospital and ED length of stay. Secondary process measures include time from ED presentation to meeting sepsis criteria and time from meeting sepsis criteria to completion of each bundle item. Table 4 presents baseline performance on a subset of clinical and process measures. The study includes balance measures to evaluate the overtreatment of patients at risk of sepsis. For example, the administration of antibiotics early in the clinical course of sepsis may not improve outcomes [53-55]. Several randomization schemes were considered to evaluate Sepsis Watch versus conventional treatment. However, expert clinicians can struggle when asked to complete a clinical task both with and without computer-aided support [56]. The intensive training of a small group of users also increased the risk of cross-group contamination. Ultimately, the single-site and operational nature of the innovation pilot made a prepost design most appropriate for this study. The data from this pilot can be used to recruit additional internal and external sites for a cluster-randomized trial to better characterize the causal relationship between Sepsis Watch and clinical outcomes [2].

**Table 4:** Baseline sepsis management performance at Duke University Hospital (n=1377).

Outcome measures	Baseline performance^a^
3-hour antibiotic compliance, n (%)	856 (62.16)
3-hour lactate compliance, n (%)	1064 (77.27)
3-hour blood culture compliance, n (%)	1237 (89.83)
3-hour antibiotic, lactate, and blood culture compliance	701 (50.91)
In-hospital mortality, n (%)	122 (8.86)
ICU^b^ requirement, n (%)	491 (35.66)
Time from ED^c^ arrival to meeting sepsis criteria (hours), median (25% percentile, 75% percentile)	1.93 (0.83, 5.08)
Length of stay in ED (hours), median (25% percentile, 75% percentile)	11.59 (9.87, 14.14)
Hospital length of stay overall (hours), median (25% percentile, 75% percentile)	125.42 (75.56, 215.18)

^a^Clinical and process measures for preimplementation cohort of adults who develop sepsis in the Duke University Hospital emergency department between March 1, 2018, and August 31, 2018.

^b^ICU: intensive care unit.

^c^ED: emergency department.

## Discussion

### Principal Findings

**Figure 4:**
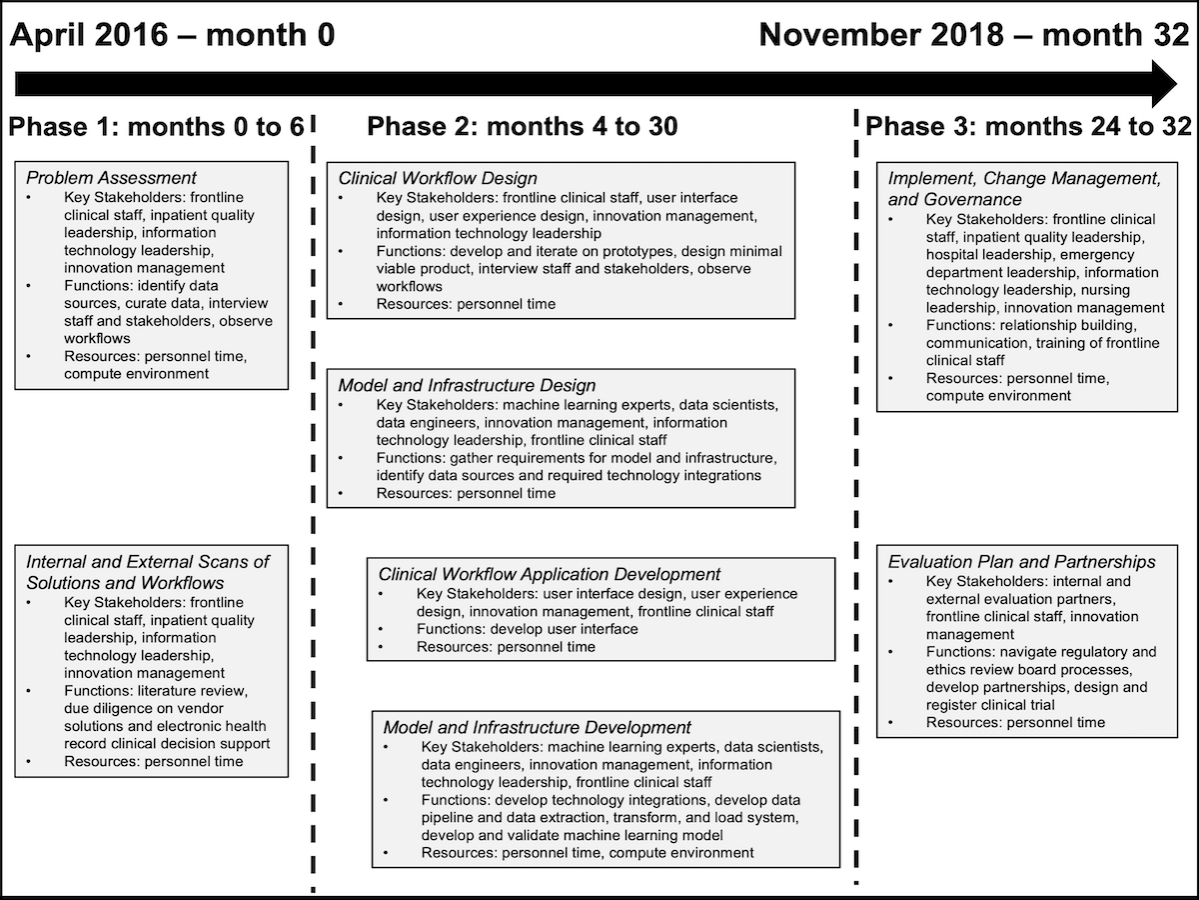
Timeline of steps involved in translation of Sepsis Watch from problem identification to integration into routine clinical care.

What began as a 12-month innovation pilot to improve sepsis management became a multiyear groundbreaking effort that built capabilities, partnerships, and infrastructure with profound organizational impact. Figure 4 illustrates a timeline of the various steps described above, providing detail into the key stakeholders, functions, and resources involved in each step. Certain stakeholders, such as frontline clinical staff and innovation managers, were involved in every step of the process. Other stakeholders, such as hospital leaders and external research partners, were crucial to specific steps along the path. As the first integration of deep learning into routine care delivery, capabilities had to be developed across domains of expertise, requiring significant collaboration and cross-training. Physicians had to learn how to develop, use, and evaluate machine learning models. Information technology leaders had to learn how to integrate, support, and maintain machine learning models. Data scientists and machine learning experts had to learn about clinical data sources and sepsis. Although specialized skills were developed and will continue to be developed across the organization as additional projects are executed, we expect that close collaboration between clinicians, data scientists, data engineers, innovation managers, and information technology leaders will remain crucial.

The largest resource required for the successful translation of Sepsis Watch into routine clinical care was personnel time. Commodity compute infrastructure was used to support data analyses, model training, and model implementation. Personnel time was estimated for internal accounting purposes at about 8000 hours. Notably, many trainees were involved, including statistics graduate students, medical students, and clinical fellows, whose effort is not included in the estimate. This effort significantly exceeded the resources used for a prior effort to implement a linear regression model, estimated at US $220,000 [8]. However, the technology platform and capabilities built through the Sepsis Watch integration continues to create value for the organization. This infrastructure now supports additional applications of machine learning and accelerates research and quality improvement efforts across the organization. Other institutions that do not have in-house capabilities across technical and clinical domains may face additional costs and barriers. This reinforces previous findings that academic medical centers may be uniquely positioned to conduct translational machine learning research [5].

The steps presented in Figure 4 are not mutually exclusive and do not proceed in a neat, sequential manner. There is an overlap between the different phases and activities from an individual step may recur at a later phase of the project. For example, although the pilot began in the DUH ED, additional iterations of problem assessment were completed to better understand opportunities to improve sepsis care in other inpatient settings. Similarly, additional iterations and improvements to the user interface and workflow were made during the first few months of the pilot. Teams building and integrating machine learning technologies into routine clinical care should be prepared to iterate, maintain, and improve products throughout the product lifecycle.

### Limitations

This study has several limitations. First, this is a single-center study, and the learnings and experience may not translate well to other settings. However, considering the lack of published evidence regarding the successful integration of machine learning into clinical care, initial case studies can be informative. Second, the integration of a deep learning model that changes clinical practice presents significant challenges with model updating. Feedback loops are created where Sepsis Watch may prompt clinical action for patients who do not ultimately develop sepsis [57]. Model retraining and updating will need to account for these feedback loops, which are an area of active research and will need to be explored in future work. Third, this study does not shed light on the predictors of sepsis and potential future directions of scientific inquiry. Fourth, this study does not present data on the clinical or economic impact of the integration of the Sepsis Watch. Analyses are underway and will be reported in future work. Fifth, this study does not demonstrate how well Sepsis Watch generalizes to care delivery settings beyond the ED. Future work will need to address generalizability both to external settings and other care units within the same hospital.

### Conclusions

Despite the limitations, the successful integration of Sepsis Watch into routine clinical care signified a *crossing the chasm* journey for Duke Health [58]. Initially, a small number of visionary clinicians and administrators were eager to use emerging technology to address an important clinical problem. As the project progressed over two years, a broader group of stakeholders became aware of the potential impact of integrating machine learning into clinical care. A new request for applications was announced a month before the Sepsis Watch launch in November 2018, and the Duke Institute for Health Innovation received a record number of machine learning proposals, of which five machine learning proposals were ultimately selected by senior leadership and launched in April 2019 [59]. In June 2019, Sepsis Watch was disseminated to EDs at the two Duke Health community hospitals. Numerous challenges were encountered during the path to integration, but a focus on improving patient care moved Sepsis Watch from concept to design to production. There is no playbook for how to integrate machine learning into clinical care, and many more successful implementations are needed to develop best practices. Learnings from the Sepsis Watch integration have informed processes designed to improve the execution of machine learning projects within our health system. These learnings can provide direction to teams pursuing machine learning integrations into care elsewhere.

## Appendix

Multimedia Appendix 1 Supplement to the manuscript including two tables.

## Abbreviations

CDS: clinical decision support
CMS: Centers for Medicare & Medicaid Services
D&S: Data & Society Research Institute
DUH: Duke University Hospital
ED: emergency department
EHR: electronic health record
ICU: intensive care unit
MGP: multitask Gaussian process
qSOFA: quick Sequential Organ Failure Assessment
RNN: recurrent neural network
RRT: rapid response team
SSRI: Social Sciences Research Institute
