# Supplementary material for: Real-World Integration of a Sepsis Deep Learning Technology Into Routine Clinical Care: Implementation Study
Source: JMIR Med Inform. 2020 Jul 15;8(7):e15182. doi: 10.2196/15182 (PMC7391165; doi:10.2196/15182)
Supplement: Multimedia Appendix 1 [file medinform_v8i7e15182_app1.docx]

**Supplemental Table 1 (ST1).** Sepsis Definition Criteria.

| **Sepsis Criteria** | **Components of Criteria (time window in hours for the “shelf life” of each criteria)** |
| --- | --- |
| 2 or more SIRS criteria |  |
|  | Temperature >38^o^ C or <36^o^ C (6 hours) |
|  | Pulse >90 (6 hours) |
|  | Respiratory Rate >20 (6 hours) |
|  | WBC count >12 x 10^9/L, <4 x 10^9/L; or %bandemia >10% (24 hours) |
| Suspicion for infection |  |
|  | Blood culture order (24 hours) |
| 1 element of end organ failure |  |
|  | Serum creatinine >2.0 mg/dL (24 hours) |
|  | INR >1.5 (24 hours) |
|  | Total bilirubin >2.0 mg/dL (24 hours) |
|  | SBP <90 or decrease in SBP>40 mm Hg (6 hours) |
|  | Platelets <100 x 10^9/L (24 hours) |
|  | Lactate >2.0 mmol/L (24 hours) |

Specifications for computable sepsis criteria used to define time of sepsis. The following criteria were used retrospectively to label sepsis cases for model training and testing and prospectively in the Sepsis Watch management user interface to identify patients who meet these sepsis criteria. Specific time window and numeric thresholds were specified and agreed upon by an internal team of interdisciplinary clinicians.

**Supplemental Table 2 (ST2):** Machine Learning Model Inputs.

| **Variable Type (n)** | **Variables** |
| --- | --- |
| Demographics (3) | Age, Gender, Race |
| Other Encounter Info (4) | Admission Source, Admission Type, Weight at admission, Prior # Sepsis Encounters |
| Comorbidities (29) | Congestive Heart Failure, Valvular Disease, Pulmonary circulation disorders, Peripheral vascular disease, Hypertension, Paralysis, Other neurological disorders, Pulmonary circulation disorders, Diabetes without chronic complications, Diabetes with chronic complications, Hypothyroidism, Renal failure, Liver disease, Chronic peptic ulcer disease, HIV and AIDS, Lymphoma, Metastatic cancer, Solid tumor without metastasis, Rheumatoid arthritis / collagen vascular diseases, Coagulation deficiency, Obesity, Weight loss, Fluid and electrolyte disorders, Blood loss anemia, Deficiency anemias, Alcohol abuse, Drug abuse, Psychoses, Depression |
| Laboratory Values (29) | Albumin, ALT, Ammonia, AST, Bandemia, Bicarbonate, Bilirubin, BUN, CK-MB, Creatine Kinase, CRP, D-Dimer, ESR, Fibrinogen, Glucose, Hematocrit, INR, Lactate, LDH, Magnesium, PCO2, pH, Platelets, PO2, Potassium, Serum Creatinine, Sodium, Troponin, WBC |
| Continuous-valued Vital Signs (7) | Systolic BP, Diastolic BP, MAP, Pulse, Pulse Oximetry, Respiratory Rate, Temperature |
| Categorical-valued Vital Signs (2) | AVPU [alert, voice, pain, unresponsive] Score (binary: alert / other), Any Supplemental Oxygen (binary) |
| Medications (10) | Antibiotics, Benzodiazepines, Chemotherapy, Heparins, Immunosuppressants, Insulins, IV Fluids, Opioids, Steroids, Vasopressors |
| Blood Culture Order Time (1) | Blood Culture (indicating suspected infection) |
| Missing Data Indicators (36) | Indicator variables for the 29 Laboratory Values and 7 Continuous-valued Vital Signs, indicating whether variable was measured in the last hour |

List of all variables included as features for machine learning model development. There are dynamic features with repeated measurements over time, including laboratory values, vital signs, medications, and missing data indicators. There are static features with fixed values for an encounter that are repeatedly fed to the model, including patient demographics, encounter information, and comorbidities.
